# Tuberculosis

**Published:** 1912-09

**Authors:** Robert Roxburgh


The Bristol

Medico-Chirurgical Journal.

"*Scire est nescire, nisi id me*
*Scire alius sciret.*"

SEPTEMBER, 1912.

### TUBERCULOSIS.

BY

Robert Roxburgh, M.B. Edin., *President.*

The Inaugural Address at the Annual Meeting of the Bath and Bristol Branch of the British Medical Association, 19th June, 1912.

It is usual for the President to inaugurate his term of office by an address, and in choosing a subject suitable for the occasion I found myself face to face with two great topics which are at present engaging the attention both of the medical profession and of the general public to a degree beyond all previous experience—topics interesting alike and of first-rate importance from the points of view of medical science, of sociology, and eugenics. I refer to *tuberculosis* and to the problem of the *feeble-minded.* The former subject is so insistent at the present time in its claims on our attention, that although medical literature teems with it in its many aspects and phases, I venture to think that a brief historical survey of the evolution of our modern treatment of pulmonary tuberculosis, with some comments on that treatment as it stands to-day, may not be out of place.

It is obvious that a consistent and appropriate method of treating any disease must be based on the conception held of its pathology. Cullen, in his *First Lines of the Practice of Physic,* published in 1774, defines phthisis pulmonalis as "an expectoration of pus or purulent matter from the lungs attended with a hectic fever." He refers its causation to five separate heads—(1) Hæmoptysis, (2) suppuration of the lungs in consequence of pneumonia, (3) catarrh, (4) asthma, and (5) tubercles. This curious etiology, which seems to us to confound symptoms with pathological entities, leads up to a special emphasis being laid on the last head, tubercle, which he pronounces much the most frequent cause of any, and which he believes to be due to an "acrimony" in the blood producing these indurations. Cullen's views of treatment were conditioned by what he regarded as the particular category to which the case belonged. "A phthisis from tubercles," he says, "has I think been recovered; but it is of all others the most dangerous, and when arising from a hereditary taint is almost certainly fatal." His treatment, based on the idea that what the physician had to do was to avoid *inflammation of the tubercles,* was a general anti-phlogistic regimen, including blood-letting and low diet, a total abstinence from animal food, and the using of vegetable food almost alone. He admitted milk somewhat grudgingly to this diet. A second element of treatment was to avoid any violent exercise of respiration, any considerable degree of bodily exercise, any position of the body which straitens (*i.e.* narrows) the capacity of the thorax, and lastly, cold affecting the surface of the body, which might determine blood to the internal parts. Blisters and issues applied to the thorax were advocated as removing determination of blood to the pulmonary vessels. He had little to say in favour of such modes of "gestation" as riding on horseback or travelling in a carriage, but he strongly recommended sailing in boats on the sea as involving a passive kind of movement and a pure air. It is not, perhaps, surprising that if Cullen's principles just described were widely propagated, as no doubt they were, a system of anti-phlogistic treatment became general in dealing with phthisis which that really great physician might in many instances himself have disapproved, and which lingered on well into the Victorian era.

The literature of the late eighteenth and early nineteenth centuries reflects the popular notions about consumption. "Going into a decline" meant being shut up in a warm room, being dedicated to hopeless invalidism, and waiting for inevitable early death.

By his penetrating genius and accurate observation Laennec unified the pathology of tubercular phthisis. While holding that pneumonia or pleurisy might precede, accompany, or follow tuberculous lesions, he maintained that tuberculosis was due to a specific constitutional affection, whose cause was then beyond reach and could not be defined, but *was not primarily an inflammation.* One of the sections of his classical treatise on mediate auscultation (second edition, published 1826) is headed "Is Phthisis Curable ?" Laennec's answer to this question seems strangely at variance with what we now regard as the most demonstrable of all pathological facts, viz. the curability of "closed cases." He declares the idea of cure in the early stages to be perfectly illusive, but that in the later stages—*after* softening of the tubercles and formation of an ulcerous excavation—cure is possible in some cases. Under medicinal or other therapeutic means used in his day he mentions (though certainly without approval) the vapours produced by sublimating zinc, lead, sulphur, etc., the air of cow-houses, that of stagnant water, the snuff of candles, the inhalation of oxygen, sulphuretted hydrogen and carbonic acid, and for internal use a multitude of substances, such as acorns (roasted or raw), different kinds of mushrooms, shell fish, frogs and vipers, millipedes, wolf's bane (one of the aconites), chocolate, sugar of roses. Other methods employed were mercurial salivation and frequently-repeated emetics.

Laennec, to his credit, condemned such empirical absurdities, while in change to a marine atmosphere he saw the most hopeful means of amelioration, and always recommended when possible a sea voyage and a residence on the sea-coast in a mild climate. He went so far as to endeavour to produce a marine atmosphere for his hospital patients in Paris by hanging up fresh seaweed in the wards!

There can be no doubt that the fatal error of the time to which we are alluding was the confusion in the medical mind on the subject of fever, so that the exhausting pyrexia due to absorption of toxins was regarded as "over-action" or *phlogiston,* to be reduced by a combination of lowering, depleting, and devitalising proceedings, which hurried most of the patients to their graves.

It is not to be wondered at that such therapeutics occasionally stirred the opposition of independent observers, and that in spite of professional fashion and prejudice more rational ideas prevailed here and there. As early as 1747 a doctor in the Highlands of Scotland addressed a printed letter to the profession in London, insisting on fresh air and diet as the essentials of successful treatment of phthisis, medicinal treatment being secondary and unimportant.

Andrew Stewart (father of the late Dr. A. P. Stewart, of Middlesex Hospital, who was the chief pioneer in distinguishing typhoid from typhus) studied theology, and was licensed as a preacher by the Presbytery of Edinburgh in 1796. He afterwards studied medicine, and became an M.D. He was presented to the parish of Erskine on the Clyde by the 10th Lord Blantyre, whose daughter he treated for consumption and married. Dr. Stewart inaugurated a method of treating consumptives by fresh air, mild exercise, driving or riding, a good but not excessive supply of digestible food, daily sponging the whole body with vinegar and water, and plunge baths or preferably the open sea. He met with much success. But when a paper descriptive of his method was read in 1825 at a medical society in Glasgow, the majority of members were hostile, declaring that almost every case of phthisis was accompanied in the early stages by inflammatory action, for which a distinctly anti-phlogistic plan of treatment was indicated. Dr. Stewart was referred to in the minutes as "a medico-clerical gentleman of consumptive notoriety." His treatment became known as the "*beef-steak and porter treatment.*"

In a treatise on domestic medicine written in 1783 by Dr. William Buchan, he advocated pure country air and riding on horseback as most important, and milk he considered as of more value in this disease than the whole *materia medica.*

Nearly a century after the Highland physician sent his letter to his friends in London, Mr. George Bodington, of Sutton Coldfield, Warwickshire, published an "essay on the treatment and cure of pulmonary consumption on principles natural, rational and successful." It is refreshing to read this sensible little work, with its outspoken condemnation of the phlogistic theories and the ruinous practice of the time. Like all reformers, he had to endure the contemptuous criticisms of the "superior persons" of his day. He insisted that the nutrient, muscular and sanguiferous systems should be maintained in the highest perfection possible, and to this end that the patient should live as far as practicable in the open air, undeterred by the state of the weather, that he should have an amount of exercise suitable to his condition, and that he should have an ample and frequent supply of nourishing and digestible food. The following quotation reveals the reception accorded to his views:—

"Sir James Clark rather sarcastically alludes to what he terms the 'beef-steak and porter' system, which he decidedly condemns, apparently guided by the phlogistic theory. I could never recommend porter and beef-steaks to any person suffering from tubercular consumption, not from any preconceived notion of 'phlogiston,' but on account of its very grossness and unfitness for a consumptive patient. On the other hand, neither could I recommend to such an one from a prejudice in favour of the aforesaid theory of 'phlogiston' a meagre diet of vegetables, rice and water, aided by tartarised antimony, etc. I should recommend to one thus consuming away under the influence of this *wasting disease* a nutritious diet of mild fresh animal and farinaceous food, aided by the stimulus of a proper quantity of wine, having regard to the general state and condition of the patient. If this is to be called 'the beef-steak and porter system,' then I am guilty of patronising it; but to my mind it rather has the character of a preservative system, whilst the wasting plan is as much entitled to be called the destructive one."

Again, referring to the inhalation of gases, he says: "The only gas fit for the lungs is the pure atmosphere freely administered without fear. Its privation is the most constant and frequent cause of the progress of the disease. . . . How little does the plan of shutting up the patient in close rooms accord with this simple and obvious principle! As to the result of such a practice, it is known to all: one-fifth of the deaths annually in England are from consumption, whilst cures are scarcely ever heard of and never expected. Despair seems to have taken full possession of the medical profession as regards this destructive disease."

Bodington was the real founder of the sanatorium system. He recommended that patients should live in a suitable house specially selected as to site and soil, and that they should be under the constant supervision of a medical man well fitted by training to adapt the treatment to each individual case. He also emphasised the importance of after-care, and of apparently cured persons avoiding city life and taking up such rural pursuits as gardening and the like. The book contained a record of more or less advanced cases greatly benefited and some seemingly cured.

The *Lancet* in 1840 reviewed the work, and its scepticism was outspoken.

Bodington was in advance of his age. His doctrines fell on ears deafened with prejudice, and it was not until nineteen years later, when Brehmer founded the great sanatorium at Görbersdorf, in Silesia, on principles identical with those of Bodington, that the sanatorium idea began to take root on English soil. A tardy honour to this reformer's memory was the republication in 1901—sixty-one years after its original issue—by the New Sydenham Society of his excellent little monograph.

In 1855 Dr. Henry MacCormac, of Belfast (father of the late well-known surgeon, Sir William MacCormac) published an essay on consumption, in which he pressed on the profession his sanguine belief in the curability of the malady if properly treated. He was a convinced and strenuous fighter for fresh air, and had little patience with what he considered the groundless fear of "taking cold." He expressed himself with Celtic fervour on this subject.

MacCormac's was the usual reward of the innovator and the reformer. When in 1862 his views were brought before the Medico-Chirurgical Society of London, one member characterised the reading of the paper as a waste of time, another asked that the Society should be protected against the reading of such productions, and the customary vote of thanks was refused. But MacCormac was not discouraged, and to the end of his days pursued the campaign.

Mention may properly be made here of Dr. John Hughes Bennett, the distinguished physiologist and Professor of Clinical Medicine in Edinburgh, to whom we owe the introduction of cod-liver oil as an addition to the diet of the strumous. He paid peculiar attention to the digestive system of the consumptive, and believed that in them an excess of acidity existed in the alimentary canal, neutralising the alkaline secretions of the salivary and pancreatic glands ; hence that carbonaceous and fatty metabolism was interfered with, and the patient consumed his own fat. Whether or not this theory accords to any large extent with proven facts, no one will deny that cod-liver oil is a valuable adjuvant to treatment. Hughes Bennett was sympathetic to the views of Dr. Stewart, of Erskine, and did not share the hopeless outlook usual in his day. His text-book of medicine in which these opinions are put forward appeared in 1859.

Sir Benjamin Ward Richardson in 1857 earnestly pleaded for the hygienic treatment of consumption.

In Germany, Hermann Brehmer, who had been influenced by Bodington's writings, started in 1854 a small sanatorium, and in 1859 founded the great institution already referred to at Görbersdorf. His assistant, Dr. Dettweiler, who had been cured there, afterwards erected the famous sanatorium Falkenstein, in the Taunus mountains. His great desire was to see suitable sanatoria for the working-classes over Germany, and this was afterwards accomplished through the State Health Insurance Law, in imitation of which our present invalid insurance provision has been to a great extent modelled.

It was largely through personal acquaintance with Dr. Walther's private institute at Nordrach, near Biberach, on the part of British medical men that the sanatorium idea became familiar to English minds, and many Nordrachs are now accomplishing the same results on British soil that have been achieved in Germany.

In America the most brilliant work is associated with the enthusiastic Trudeau and his co-workers, Drs. Kinghorn, Lawrason Brown and Baldwin, at the Adirondack Cottage Sanatarium, Saranac Lake, whose reputation is world-wide.

There are at this moment some ninety special sanatoria and hospitals for tuberculous disease in this country, exclusive of workhouse infirmary wards set apart for this purpose, and very few people will be found to deny the extreme value of these institutions. The limitations of their usefulness are due (1) to their utter insufficiency in number to cope with more than a fraction of the diseased population, (2) to the fact that, save in only one or two instances, they have to exclude the very class that most urgently need their services, namely those who are too poor to pay anything for their maintenance, and (3) to their inability to keep the patients long enough under care for anything like actual arrest of the mischief. This, of course, refers to the charitable, and not to the private institutions. As we stand at present, the most important work of the sanatoria is not so much curative as educative.

Statistics of actual complete recovery in these institutions are extremely meagre, but all must admit the great improvement so often recorded and visibly demonstrated, and, above all, the hygienic habits inculcated on the inmates, and we must hope continued to a large extent at home after the patients' return. And while speaking of education, I would urge that all school authorities without exception should require that children, both in primary and secondary schools, be taught as a compulsory subject the foundation truths of physiology, and especially the care of the teeth, the skin, and the alimentary tract. The steady decline in phthisis which has been taking place in our country since the year 1855, accentuated after the *Public Health Act* of 1875, again after the discovery of the tubercle bacillus in 1882, still more after the passing of the *Housing of the Working Classes Act* of 1890, and most of all after the establishment of sanatoria and the beginning of notification (about 1899), this decline will surely hasten more and more rapidly when public opinion is fully enlightened, when spitting in public places is a penal offence, and when the social conditions and the habits of the labouring classes have reached a higher level.

This is not the place to discuss the National Insurance Act of 1911, or the relation of the medical practitioner to its working; but this at least must be said, that the scheme for dealing with tuberculosis recommended in the Interim Report of the Departmental Committee, issued only a week or two ago, ought, if thoroughly carried out, to mark a great step forward towards the goal at which we all aim, the total extinction of the disease, and is at least the first attempt to deal with the problem on a national scale.

Before concluding my remarks on the history of treatment, I must allude to a subject much too large for more than a passing reference, but one of the greatest therapeutic importance. I mean the use of tuberculin in the treatment of pulmonary tuberculosis.

After Koch's rather premature introduction in 1891 of what is known as "old tuberculin" as an active immunising agent, and the unsatisfactory and even disastrous consequences that too often followed his dosage, the remedy fell into disrepute, and it was quite ten years before it began to be rehabilitated in the estimation of professional men, a process much stimulated by Sir A. Wright's discovery of opsonins and their measurement and his work on vaccines. Such workers, however, as E. L. Trudeau, R. W. Philip, and many others had continued to study the subject, to try various preparations and methods of dosage, and to watch and record results.

Very diverse are the views of its efficacy among experts, but nearly all are agreed that though no true specific, it is in suitable cases a definite aid to curative treatment. A few go so far as to regard it as of more importance than all other factors of treatment, and "tuberculin dispensaries" for its systematic use already exist among us. This diversity of opinion is largely owing to the impossibility of standardising the remedy, to the facts that slight differences of manufacture or a few weeks of age markedly alter its potency, and that no decision has been generally reached as to which of the numerous preparations is the most reliable, while accurate statistics of its curative activity are *ipso facto* impossible of attainment. The expert workers, however, are very sanguine that before long better tests of its action and regulations of procedure may be formulated. When the opsonic index was first worked out for various toxins it was used as a guide in the administration of tuberculin. It is so no longer. The "intensive" method is now generally practised. A minimal dose—about the $\frac{1}{10000}$ or a little more of a milligram—of solid bacterial matter is first used, and is very gradually increased in doses repeated at intervals of two to five days until the earliest signs of reaction appear. The injection is now intermitted until the reaction has subsided, when slightly increased dosage at wider intervals is continued. Doses of 1, 2 or even 3 milligrams may ultimately be reached with the augmented toleration of the patient.

Trudeau is worth quoting here. He says: "The first impression which the physician receives who begins to make use of tuberculin is a most profound respect for the tremendous potency of this toxin. A toxic substance which in so infinitesimal a dose as the one five-thousandth of a milligram of the solid substance contained in Koch's bacillary emulsion may produce typical and marked constitutional disturbance in the tuberculous individual is certainly not to be used heedlessly and is potent for evil if carelessly administered. I would not urge any physician who prizes his peace of mind to embark on the treatment of tuberculosis by this method unless he is prepared to begin with minute doses and increase with the utmost caution."

While Koch thought pronounced reactions were necessary to success in this form of immunisation, Trudeau holds strongly that fever reactions are not necessary for good results, and in fact should be strenuously avoided.

At the Royal National Hospital at Ventnor one milligram is the highest dose yet reached. Dr. Gilchrist, the senior resident medical officer there, informs me that on the whole the patients desire the treatment, and frequently express themselves as feeling better after the dose, *i.e.* after twenty-four hours have passed. Several men at Ventnor who are going through a course of graduated work have also been having tuberculin, and almost always have been able to go on with their work as soon as the temperature (with a febrile reaction) has returned to normal. At Winsley during 1911, we learn from Dr. Crossley's report, out of 269 patients admitted 42 were treated with tuberculin; of these 19 did very well, 9 fairly well, 2 made no improvement, and in 12 the treatment could not be continued. The method of dosage was that already described.

Dr. Thurnam at Nordrach-on-Mendip uses tuberculin as a routine treatment in all suitable cases. He considers that each different preparation has a special efficacy for the different individual case, and has to be selected experimentally.

The bacillary emulsion, containing triturated bacilli with both endo- and exo-toxins, is the form of tuberculin now most generally used, and at Saranac is almost exclusively employed. But all tuberculins seem to depend for their potency on the same substance, and all act in the same way, immunity to one kind carrying immunity to others.

On the other hand, Dr. Marcus Paterson, as is well known, uses no tuberculin at all at Frimley, the great sanatorium connected with the Brompton Hospital, preferring the auto-inoculation method of supplying antigen to the blood by means of carefully-regulated exercise, and theoretically his position is perfectly logical.

As regards the selection of cases, all are agreed that early and afebrile ones are most hopeful; but, tuberculin or no tuberculin, these are the cases that always respond best to rational treatment. There is, however, an interesting consensus of opinion on another class of case which deserves to be mentioned.

Dr. Edward Squire, the well-known authority of the Mount Vernon honorary staff, writes to me: "Some patients after progressive improvement for some weeks or months under sanatorium treatment get to a stage where improvement stops; there they stick fast. In such cases tuberculin often helps them on."

In the *Johns Hopkins Hospital Bulletin* for August, 1909, Drs. Hamman and Wolman, reporting on their work with tuberculin at the Phipps Dispensary at Baltimore, write: "Perhaps the most striking improvement was attained in cases of a moderately or far advanced character which had remained stationary after experiencing some amelioration from general hygienic and dietetic treatment." Again, in a private letter from Dr. Kinghorn, of the Adirondack Cottage Sanatarium at Saranac, he says: "There are a group of cases which we see at the health resorts which have chronic lung tuberculosis. Their physical condition and the lung condition seem to change little or not at all from year to year. I have repeatedly been able to carry some of these cases to cure or arrest of the disease by tuberculin treatment."

The rationale of these facts seems clear enough. In so quiescent a condition too little toxin was produced by the organism to stimulate the defensive powers of the body cells, and a supply of toxin from without produced the requisite stimulus. This is not the place to refer to the use of tuberculin in other situations of infection—lymphatic glands, skin, genito-urinary system, joints, etc.—but in these there is no doubt a great field for this remedy.

I have now traced the treatment of phthisis from a century and a half ago to the present day. The outlook is full of hope. If we remember that the death-rate from phthisis in England and Wales, so far as statistics are available, has declined from 39·9

per 10,000 persons in 1838 to 11.5 in 1906, and that it dropped 14 per cent. from 1901 to 1909, and in London in the same period no less than 33 per cent., we may well hope that in two generations the "white scourge" will have become only a memory in this land, like typhus or leprosy.

Towards this end a national specific education is required; over-crowding must cease, and the most vigorous application of preventive measures must be adopted to stamp out bovine tuberculosis, to secure a pure milk supply, and to guard our shores from the incursion of phthisical persons from abroad.

